# Impact of proactive nurse participation in ICU family conferences: a mixed-method study

**DOI:** 10.1186/2197-425X-3-S1-A929

**Published:** 2015-10-01

**Authors:** M Garrouste-Orgeas, A Max, C Grégoire, S Ruckly, M Kloeckner, S Brochon, E Pichot, C Simons, M El Mhadri, C Bruel, F Philippart, J Fournier, K Tiercelet, J-F Timsit, B Misset

**Affiliations:** Saint Joseph Hospital Network, Intensive Care, Paris, France; University Paris VII Denis Diderot, IAME, UMR 1137, Paris, France; Outcomerea, Paris, France; University Paris V Paris Descartes, Paris, France

## Introduction

Few studies have assessed the impact on everyday medical decisions of better communication, most notably delivered by a multidisciplinary team.

## Objectives

To investigate family perceptions of having a nurse participate in family conferences and to assess the psychological well-being of the same families after ICU discharge.

## Methods

We designed a mxed-method design with a qualitative study embedded in a single-center randomized study. The design was a parallel-group randomized trial comparing family conferences with versus without the proactive participation of a nurse in family conferences. All physicians and nurses received training with the use of role players. A standard conference guide was created for different situations (admission, weekly, end-of-life). We included one family member for each consecutive patient who received more than 48 hours of mechanical ventilation in the ICU. Each family member fulfilled the Peri-Traumatic Dissociation Questionnaire (PRDQ) at ICU discharge, the Impact of Event Scale-revised (IES-R) and the Hospital Anxiety and Depression Scale (HADS) at day 90. Each interview at ICU discharge was transcribed verbatim and evaluated using interpretative phenomenological analysis.

## Results

172 family members were eligible, and 100 (60.2%) family members were randomized; among them, 88 underwent semi-structured interviews at ICU discharge and 86 completed the Peritraumatic Dissociative Experiences Questionnaire (PDEQ) at ICU discharge then the Hospital Anxiety Depression Questionnaire and the Impact of Event Scale (for posttraumatic stress-related symptoms) 3 months later. At ICU discharge, the median [IQR] PDEQ was not significantly different between the control and the intervention group (14.5 [11-23] vs 13 [10-17], p = 0.17). The median [IQR] IES-R score was not significantly different between the control and intervention groups (24 [12.5-45] and 21 [9-23], respectively; p = 0.24). The intervention and control groups were not significantly different regarding the prevalence of posttraumatic stress-related symptoms (52.3% vs. 50%, respectively; p = 0.83), anxiety symptoms (52.3% vs. 33.3%, p = 0.08), or depressive symptoms (38.6% vs. 23.8%, p = 0.14).

The qualitative data indicated that the families valued the principle of the conference itself. In the intervention group, perceptions of nurse participation clustered into four main themes: trust that ICU teamwork was effective (50/88, 56.8%), trust that care was centered on the patient (33/88, 37.5%), trust in effective dissemination of information (15/88, 17%), and trust that every effort was made to relieve anxiety in family members (12/88, 13.6%).

## Conclusions

Families valued the conferences themselves and valued the proactive participation of a nurse. These positive perceptions were not associated with decreases in measures of post-ICU burden.

## Grant Acknowledgment

The Fondation de France has funded the study
